# Soybean Yield Preharvest Prediction Based on Bean Pods and Leaves Image Recognition Using Deep Learning Neural Network Combined With GRNN

**DOI:** 10.3389/fpls.2021.791256

**Published:** 2022-01-13

**Authors:** Wei Lu, Rongting Du, Pengshuai Niu, Guangnan Xing, Hui Luo, Yiming Deng, Lei Shu

**Affiliations:** ^1^College of Artificial Intelligence, Nanjing Agricultural University, Nanjing, China; ^2^College of Agriculture, Nanjing Agricultural University, Nanjing, China; ^3^College of Engineering, Michigan State University, East Lansing, MI, United States

**Keywords:** yield prediction, phenotyping, germplasm innovation, soybean, *in situ*

## Abstract

Soybean yield is a highly complex trait determined by multiple factors such as genotype, environment, and their interactions. The earlier the prediction during the growing season the better. Accurate soybean yield prediction is important for germplasm innovation and planting environment factor improvement. But until now, soybean yield has been determined by weight measurement manually after soybean plant harvest which is time-consuming, has high cost and low precision. This paper proposed a soybean yield in-field prediction method based on bean pods and leaves image recognition using a deep learning algorithm combined with a generalized regression neural network (GRNN). A faster region-convolutional neural network (Faster R-CNN), feature pyramid network (FPN), single shot multibox detector (SSD), and You Only Look Once (YOLOv3) were employed for bean pods recognition in which recognition precision and speed were 86.2, 89.8, 80.1, 87.4%, and 13 frames per second (FPS), 7 FPS, 24 FPS, and 39 FPS, respectively. Therefore, YOLOv3 was selected considering both recognition precision and speed. For enhancing detection performance, YOLOv3 was improved by changing IoU loss function, using the anchor frame clustering algorithm, and utilizing the partial neural network structure with which recognition precision increased to 90.3%. In order to improve soybean yield prediction precision, leaves were identified and counted, moreover, pods were further classified as single, double, treble, four, and five seeds types by improved YOLOv3 because each type seed weight varies. In addition, soybean seed number prediction models of each soybean planter were built using PLSR, BP, and GRNN with the input of different type pod numbers and leaf numbers with which prediction results were 96.24, 96.97, and 97.5%, respectively. Finally, the soybean yield of each planter was obtained by accumulating the weight of all soybean pod types and the average accuracy was up to 97.43%. The results show that it is feasible to predict the soybean yield of plants *in situ* with high precision by fusing the number of leaves and different type soybean pods recognized by a deep neural network combined with GRNN which can speed up germplasm innovation and planting environmental factor optimization.

## Introduction

Soybean is an important source of high quality protein and oil in the world, which contains about 42% protein, 20% oil, and 33% carbohydrate (Zhang et al., 2001). Soybean protein can enhance the body's immune function and play an important nutritional role in human tissues and cells. Soybean production for 2020 totaled 4.14 billion bushels, up 16% from 2019 in America due to a higher average soybean yield (Alsajri et al., 2020). Soybean yield prediction is of great importance to global food production, which is a highly complex trait determined by multiple factors such as genotype, environment, and their interactions. Accurate soybean yield prediction is important for germplasm innovation and planting environment factor improvement. Many researchers have tried to clarify the phenotype (such as yield) as an explicit function of the genotype (G), environment (E), and their interactions (G × E). In fact, the selection of individuals with good genotypic effect can further improve the yield of existing soybean varieties, which is also of great significance for high-yield soybean breeding. So, the earlier the prediction during the growing season the better. But until now, soybean yield has been popularly determined by manual weight measurement after soybean plant harvest which is time-consuming, expensive, and imprecise.

In recent years, digital image processing combined with machine learning technology has been applied for crop yield prediction in literature. The relationship between grain area and weight was studied using an image processing method (Zhao et al., 2019). A citrus fruit crop prediction algorithm based on color difference of citrus fruit and leaves was studied in citrus trees (Dorj et al., 2017). A region growing algorithm was proposed to segment cotton bolls into color images and count them and predict yield (Sun et al., 2019). Algorithms that rely on feature extraction (SVM, NN, RF) and algorithms that do not need feature extraction (GoogLeNet, VGG-16) were compared. The study found that the VGG-16 algorithm could effectively distinguish corn and soybean (Flores et al., 2020). In addition, a multi-rotor UAV system was developed to obtain high-resolution images and information related to geographical location, shooting angle, and environmental illumination, so as to carry out effective agricultural detection (Zhu et al., 2019).

More recently, deep neural networks have been employed in crop yield prediction, including the convolutional neural network (CNN), faster region-convolutional neural network (Faster R-CNN), single shot multibox detector (SSD), and You Only Look Once (YOLO), etc. The features of hyperspectral and color images was used to classify corn and estimate corn yield by CNN (Yang et al., 2021). Faster R-CNN has been modified for detection and yield estimation of fruits (mangoes, pomegranates, tomatoes, apples, and oranges) (Behera et al., 2021), which has also been used in prediction of melon yield (Zhao et al., 2017). Comparation of Faster R-CNN and SSD in citrus counting and yield prediction has been carried out (Qin et al., 2021). A convolutional neural network combined with linear regression was used in sorghum spike identification and weight prediction (Zannou and Houndji, 2019). Lightweight YOLO was applied to predict the yield of oil palm fruit based on images collected by UAV (Junos et al., 2021). The traditional rectangular bounding box in YOLOv3 was replaced as a circular rectangular box for better positioning of tomatoes (Liu et al., 2020). A deep neural network was designed to study the influence of genotype, environment, and their interaction on yield prediction (Khaki and Wang, 2019). There are also studies using satellite images to predict small yield using machine learning methods such as the Gaussian process regression algorithm (Sharifi, 2021).

Existing deep learning detection targets such as strawberries (Yu et al., 2019), tomatoes (Hu et al., 2019), apple (Tian et al., 2019), pepper (Hespeler et al., 2021), etc. are obviously different from the background leaves and branches of plants, which brings convenience for fruit identification due to remarkable color difference. Although the color of cucumbers (Mao et al., 2020) and corn (Jin et al., 2018) ears are similar to the leaves and vines, they have large size, small number, and low density, which also reduces the difficulty of identification. In the yield prediction of wheat (Yang et al., 2019) and rice (Crisóstomo de Castro Filho et al., 2020), the dense clustering of wheat and rice grains increases the difficulty of detection, but fortunately, the leaves shield the ears of wheat and rice slightly. A mature soybean phenotype measurement algorithm called soybean phenotype measure-instance segmentation was proposed to calculate pod length, pod width, stem length, seed length, and seed width based on PCA combined with CNN (Li et al., 2021). For soybean yield detection, leaves and pods have similar color, pods are blocked by numerous leaves, moreover the pods are clustered together, which creates a huge challenge for soybean yield forecast. Moreover, the types of pods must also be identified at the same time to predict soybean production accurately because the number of grains in different types of pods are different.

Deep neural networks, unlike early shallow neural networks with a single hidden layer, have multiple hidden layers which can effectively reveal the underlying unknown and highly non-linear relationship between the input data and output variables (LeCun et al., 2015), which have been widely employed in face recognition, automatic driving, etc., but they also require more hardware and time consumption. Generally, more neural network layers and nodes means the network is more powerful and paradoxically needs more time and hardware to run.

After comparing several mainstream deep neural networks, the YOLOv3 algorithm was chosen to recognize soybean pods and leaves in this paper. Moreover, in order to further improve the detection performance of the neural network, structure improvements were made to YOLOv3 to achieve accurate recognition of large leaves and small pods simultaneously. In addition, a generalized regression neural network (GRNN) model was established for prediction of seed number in a soybean plant by using the cumulative results of leaves and different type pods of four images taken at 90-degree intervals from different directions. Finally, soybean plant production was calculated based on the average grain weights of different type pods, which provided a new method and solution for soybean phenotype detection and germplasm innovation acceleration.

The remainder of this paper is organized as follows. Section Materials and Methods describes the materials and data used in this research and offers a detailed description of our improved deep neural network for soybean pods and leaves prediction, and yield modeling as well. Section Results and Discussion presents the results of our algorithm and models. Finally, the conclusion is given in section Conclusion.

## Materials and Methods

**Figure 1:**
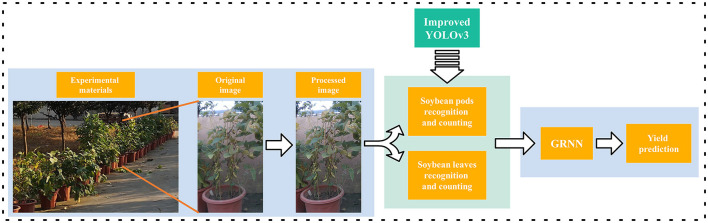
Flowchart of the soybean yield prediction method.

In this paper, the soybean yield prediction method included three steps as shown in Figure 1. First, the original imaging data of soybean plants collected were preprocessed by filtering and enhancement. Then the improved YOLOv3 model was used to identify and count leaves and different type soybean pods including one bean pods, two bean pods, three bean pods, four bean pods, and five bean pods. Finally, a GRNN model for predicting soybean yield was established based on the numbers of leaves and all categories of pods as inputs.

### Materials and Image Sampling

In this study, soybean plants were grown in pots in Wanjiang Experimental Station of Nanjing Agricultural University where the latitude and longitude are 118.62°E and 31.54°N, respectively. Each pot had four soybean plants which were evenly distributed in the pot. There were 90 pots, 360 plants, and 24 varieties in total (Xudou-18, Heidou-2, Erzaohuang, Qiyuehuang-1, Bayuehuang-4, Qingyuanxiaoqingdou, Fengdudahuangdou, Enwangheidou, Kaijiangdongdou, Huazhouhuangdou, Ganyulianmaoshao, Liyangmaojiajia, Andingxiaoheidou, Nannong1606, P06, P12, P23, P25, P53, P59, P65, GS171761, GS71244, GS71411) used for the study. All potted soybean plants were at the pod-setting stage during image sampling.

A camera (model: Intel RealSense D435 manufactured by Intel) was employed to capture soybean plant images at three different time periods (6:00–7:00, 13:00–14:00, 17:00–18:00) in order to realize the completeness of images in different light intensity environments. The camera was 1.2 m above the ground and 1.5 m horizontally from the target while image capturing. Each pot of soybean plants was photographed in four different directions at intervals of 90 degrees, so a total of 360 images were collected.

Data processing was performed on a computer with a Win10 operating system, Intel(R) Core(TM) i7-8750H processor, 8GB memory, and a NVIDIA GeForce GTX 1050 Ti display adapter. The open source deep learning framework Tensorflow2.0 was used to establish models.

### Soybean Pods and Leaves Recognition

#### Image Processing

**Figure 2:**
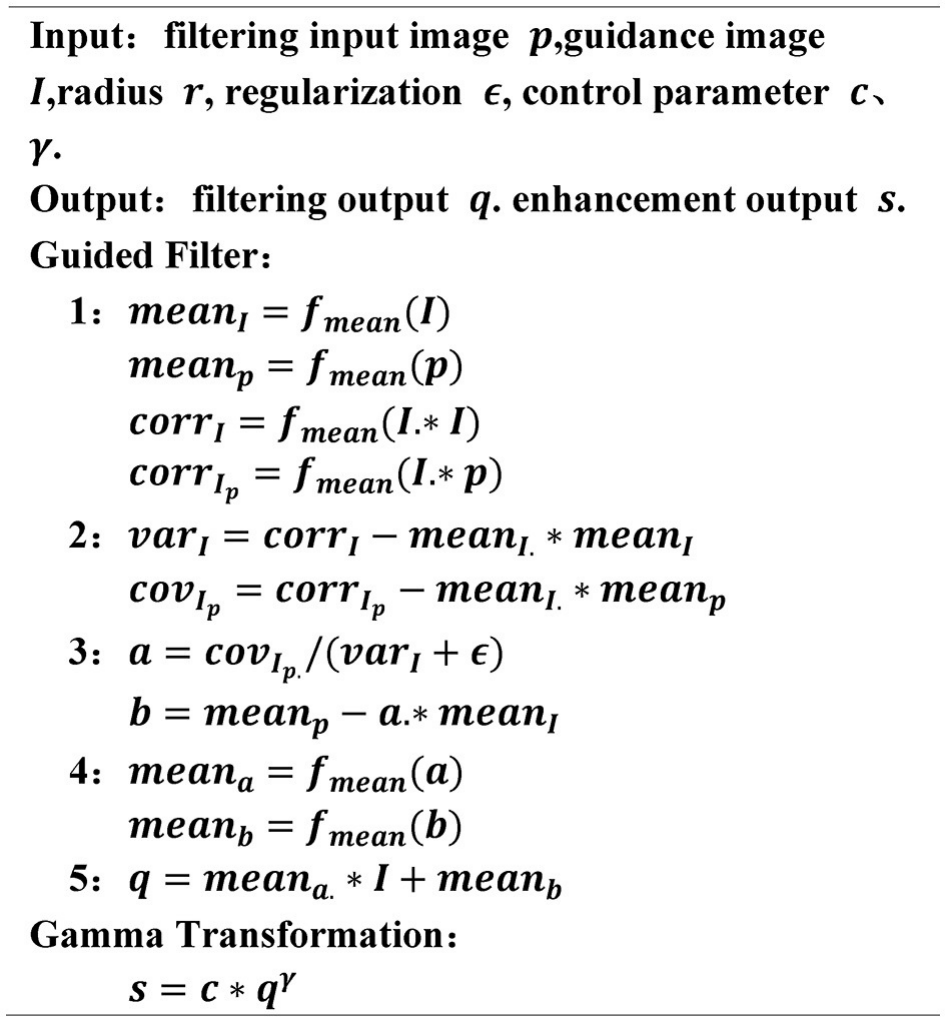
The pseudocode of denoising and enhancement algorithms.

In order to extract clear soybean plant images, denoising and enhancement treatments were carried out on soybean plant pictures due to the light environment and haze which blurred the pictures. First, *guide filter* was selected for denoising images after comparing them with the *bilateral filter* (Yu et al., 2020) and *DWT* (Rai et al., 2012) algorithms. Then, the *gamma* algorithm was chosen for image enhancement by contrast with *laplus* (Bhairannawar et al., 2017) and *log* (Maini and Aggarwal, 2010) algorithms. The pseudocode is shown in Figure 2.

**Figure 3:**
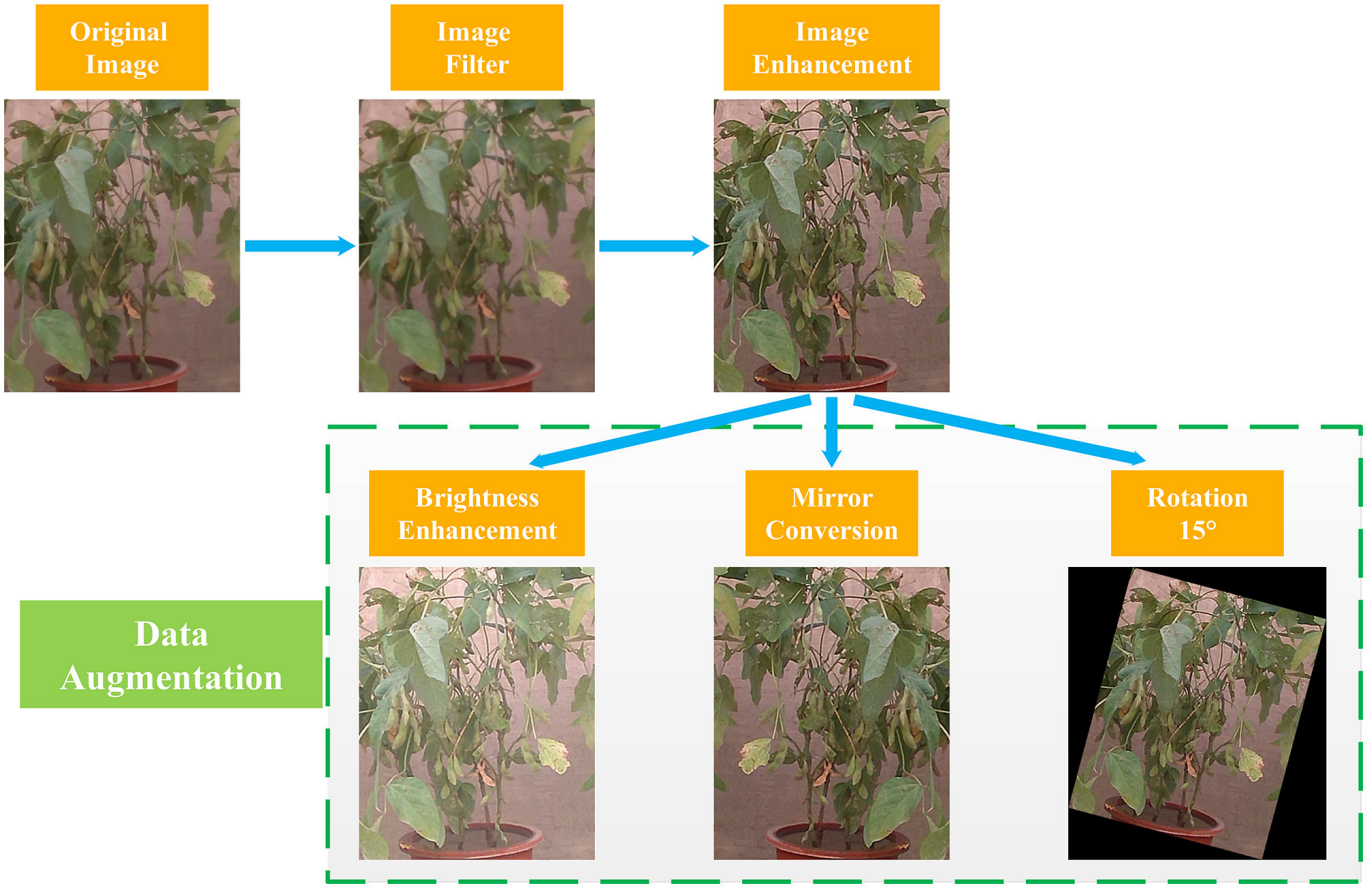
Image processing flowchart.

Since deep neural networks require a certain number of training sets to improve the accuracy of the model, a data augmentation method, a common technique in deep learning research, was employed to increase the number of existing photos to 1,800 images by rotation, scaling, mirroring, random brightness increases/decreases, and other methods. The 1,800 images were randomly divided into the training set and test set according to the ratio of 4:1, which were then used as the dataset of target detection models. The image processing process is shown in Figure 3.

#### Soybean Leaves, Pods, and Types Recognition

With the rapid improvement of the computing power of computers, deep learning has made tremendous progress, and a lot of target detection algorithms based on deep learning have been proposed. Popular deep learning algorithms such as Faster R-CNN (Ren et al., 2016), feature pyramid network (FPN, Lin et al., 2017a), SSD (Liu et al., 2016), and YOLO (Redmon et al., 2016) have been applied in different areas and show very superior performance. So, these algorithms were utilized in soybean pod and type recognition in the paper.

The detection of Faster R-CNN includes two steps, the first step is region proposal network (RPN). Features of a picture are extracted by a VGG-16 (Simonyan and Zisserman, 2014) neural network, followed by foreground background classification and first prediction of the coordinate of the generated anchor. In the second step, candidate boxes with higher confidence are selected and sent to the back of the network for the second prediction of category and coordinate values, so as to predict the specific category. Faster R-CNN has extremely high prediction accuracy but is time-consuming for training (Benjdira et al., 2019).

The feature pyramid network (FPN) is proposed to alleviate the problems of multi-scale and small target detection which selects ResNet50 (He et al., 2016) as the feature network, and fuses the high-level features with the low-level features through the up-sampling process. Moreover, layers of the same class have horizontal connections and each layer is predicted independently. Therefore, the network has more abundant features. In addition, the idea of multi-scale detection is introduced into the RPN, and the anchor frame can be generated in different scales to cover different sized objects. FPN has good accuracy and precision but again, is more time-consuming.

In the single shot multibox detector (SSD) model, there is only one detection process step. The model uses VGG as the feature extraction network, and uses multiple feature layers to predict the target. As a result, the whole model is more lightweight with both good detection accuracy and speed.

The design concept of YOLO and SSD is similar, both are one stage target detection algorithms for reaching fast performance. In YOLOv3 (Redmon and Farhadi, 2018), a 28M DarkNet-53 is employed in which its parameters are only half that of ResNet101 (Lin et al., 2017b), but the performance is close to it. Pre-clustering of YOLOv2 (Redmon and Farhadi, 2017) is inherited in YOLOv3 on the anchor frame for targets clustering in the data using nine anchor frame scales that mostly fit the targets. Moreover, the feature processing is carried out in three different levels by introducing the multi-scale concept of FPN. And multiple binary classifiers are used in the calculation of classification loss to avoid competition within the class.

**Figure 4:**
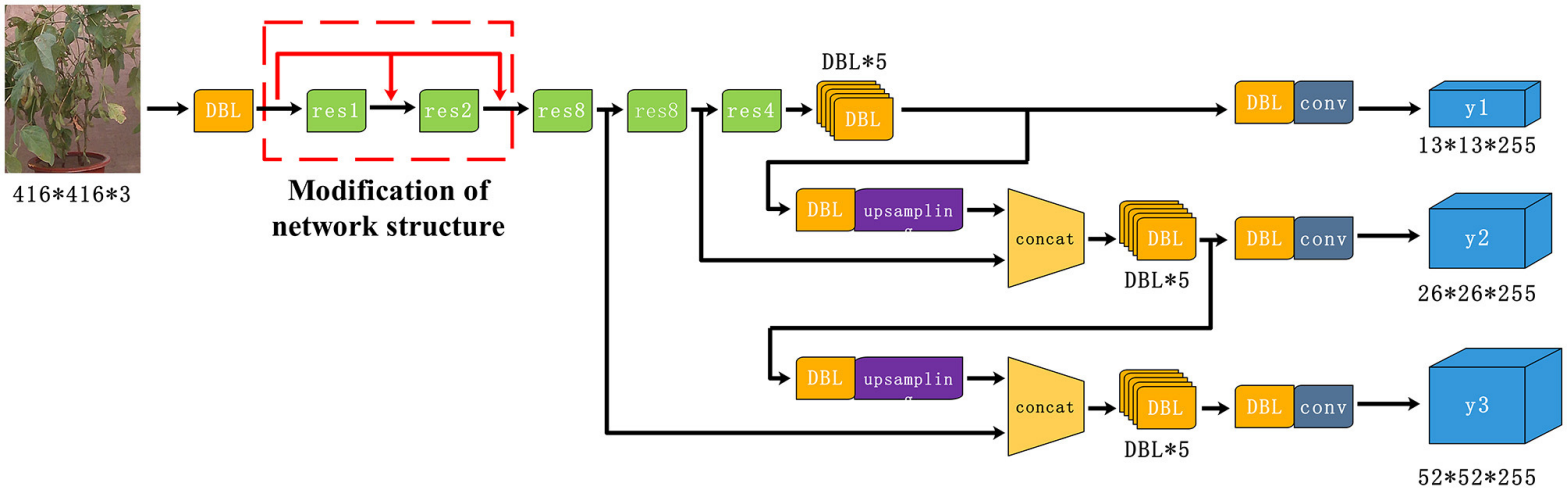
Improved YOLOv3 for soybean leaves, pod, and type identification.

There are some problems such as error detection and missing detection by using YOLOv3 for soybean leaves and pods recognition because the color of the pods is similar to the leaves and the detection performance of YOLOv3 for small target like pods is not ideal. According to the above problems, an improved approach of YOLOv3 was proposed by changing the network structure and changing the clustering algorithm for increasing prediction without sacrificing too much speed. Firstly, the feature map after one down-sampling session is superimposed to the input of the second and third residual blocks so as to increase the detail information in the deep feature map for detection. The improved network of YOLOv3 is shown in Figure 4. DBL stands for Darknetconv2D_BN_Leaky, and resn stands for the number of Res_units contained in the Res_block.

In the target detection algorithm, the anchor frame can help the model to fit the coordinate points of the target, so that the positioning task can be transformed from finding the position of the target in the image to learning the coordinate offset of the anchor frame relative to the target. In consideration of the fact that the traditional K-means algorithm is easy to implement, but the initial clustering center needs to be set artificially, in addition, different centers have great influence on the clustering results, the K-means++ algorithm (Arthur and Vassilvitskii, 2006) was selected to cluster the length and width of the anchor frame to make it fit the soybean pod and leaves better. The clustering used Euclidean distance as a metric and set *k* from 6 to 11 step by step. Because a large value of *k* reduced the convergence rate of the model, k was set to 9 in this study after several attempts. The clustering centers obtained after the convergence of the model were: (23,31), (27,21), (41,52), (64,42), (78,90), (76,103), (91,94), (106,97), and (113,107).

In terms of loss function, traditional YOLOv3 uses IoU Loss, which is composed of coordinate regression loss, confidence loss, and classification loss, and its calculation formula is shown in Equation 1.


(1) $$\begin{array}{l} L_{IoU}=1-IoU \end{array}$$

**Figure 5:**
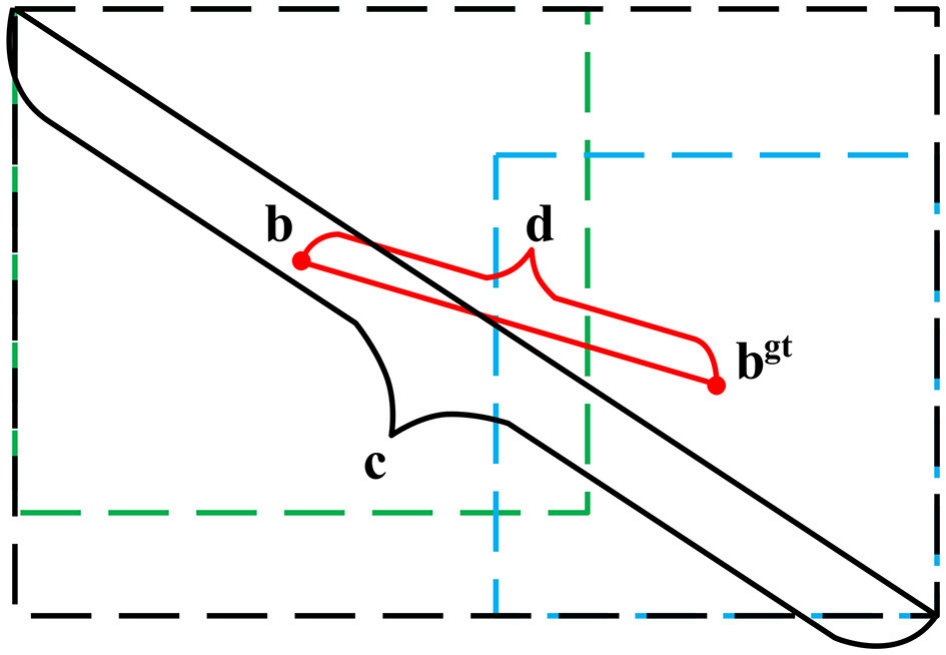
Schematic diagram of DIoU.

IoU Loss has the characteristics of scale invariance. Although IoU Loss is more advantageous than mean square error, when the relationship of the prediction box and the real box is contained and being contained, IoU Loss will be a fixed value, which has great influence on the detection effect. When the two do not intersect, the value of IoU Loss is 0, which cannot be optimized. According to the above problems, DIoU Loss (Distance IoU Loss) was used to replace IoU Loss in traditional YOLOv3, which made the regression of the target box more stable. The schematic diagram is shown in Figure 5.

DIoU is defined as,


(2) $$\begin{array}{l} DIoU=IoU-\frac{\rho^{2}\left(b,b^{gt}\right)}{c^{2}} \end{array}$$

where *b* is the central coordinate of the prediction box, *b*^*gt*^ is the center coordinate of the real box, ρ is the Euclidean distance of the two center points, and *c* is the diagonal length of the minimum outer rectangle of the two target bounding boxes.

The final definition of the loss function DIoU Loss is shown in Equation 3.


(3) $$\begin{array}{l} L_{DIoU}=1-DIoU \end{array}$$

In this paper, the improved YOLOv3 model was used to train the enhanced dataset. The size of network input was 416^*^416^*^3. The batch size was 64, the value of subbatch was 16, the momentum of dynamic parameter was 0.9, the maximum number of iterations was 14,000, the learning rate strategy was step decreasing, the initial value was 0.001, the scale parameter was 0.1, and the two step values of learning rate change were 11,200 and 12,600, respectively.

### Soybean Yield Prediction

**Figure 6:**
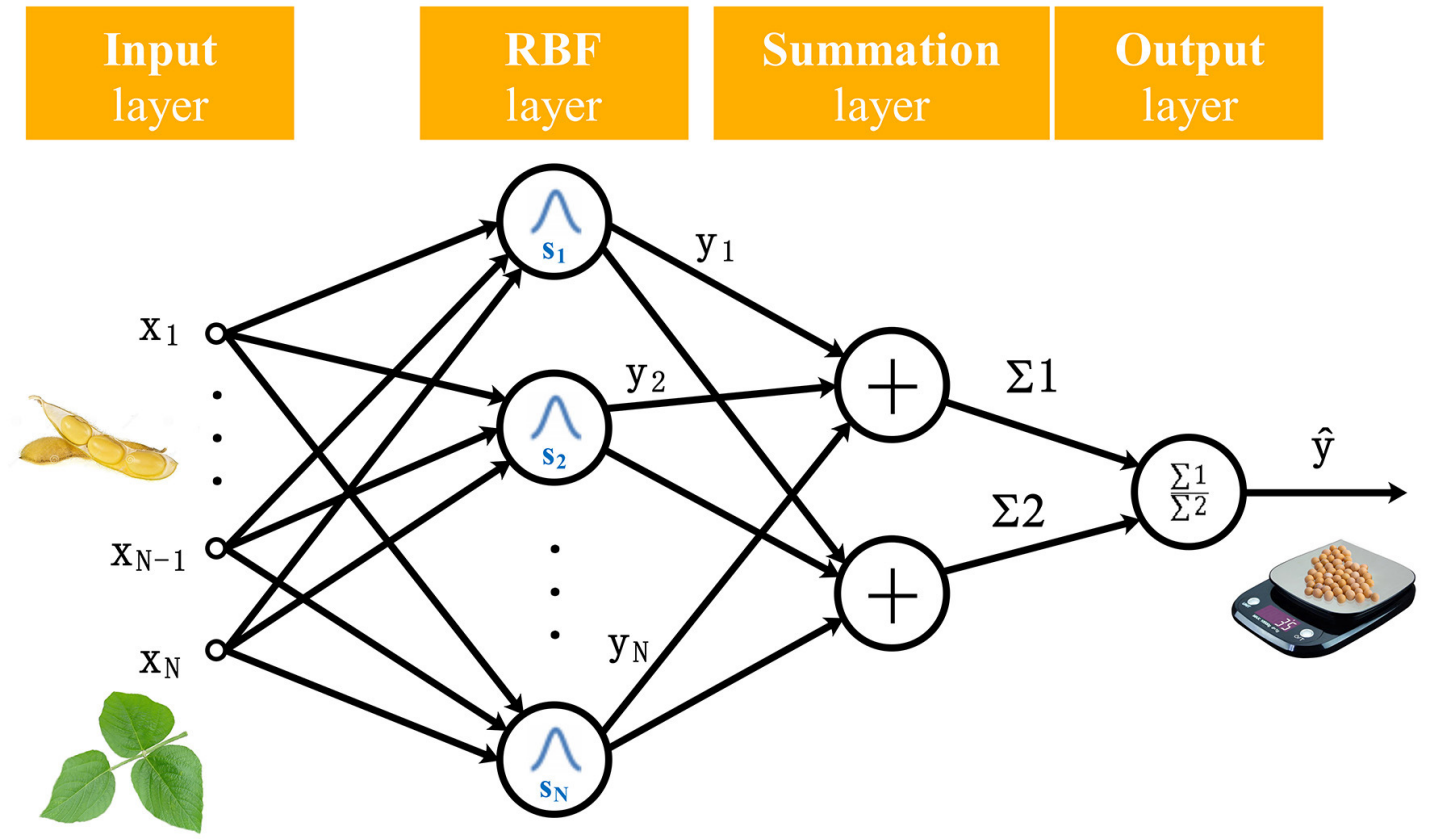
Architecture of soybean yield prediction model of GRNN.

In the soybean yield prediction task based on plant images, the counting accuracy of pods was affected because some pods were occluded by leaves, and the density of leaves was positively correlated with the number of occluded pods. In addition, pods were divided into several types due to the difference number of seeds in them. The improved YOLOv3 model was used to identify the number of leaves and different types of pods, then PLSR (Geladi and Kowalski, 1986), BPNN (Hecht-Nielsen, 1992), and GRNN (Specht, 1991) models were established respectively to predict the amount of seeds. Among them, GRNN proposed by Specht has a strong non-linear mapping ability and learning speed which is an improved technique in neural networks based on non-parametric regression. It can even obtain good prediction accuracy but only requires a small number of datasets (Izonin et al., 2021). Moreover, the network can also handle unstable data in the inputs, which is suitable for soybean yield prediction. The architecture of GRNN is shown in Figure 6.

The mathematical representation is as below,


(4) $$\begin{array}{l} Y\left(x\right)=\frac{\sum_{k=1}^{N}y_{k}K\left(x,x_{k}\right)}{\sum_{k=1}^{N}K\left(x,x_{k}\right)} \end{array}$$

where input *x* includes the numbers of soybean leaves and different type pods, *Y(x)* is the predicted value of soybean yield, *y*_*k*_ is the activation weight for the pattern layer neuron at *k*, and K(*x, x*_*k*_) is the radial basis function kernel (Gaussian kernel) as formulated below.


(5) $$\begin{array}{l} K\left(x,x_{k}\right)=e^{-d_{k}/2\sigma^{2}} \end{array}$$

(6) $$\begin{array}{l} d_{k}={\left(x-x_{k}\right)}^{T}\left(x-x_{k}\right) \end{array}$$

where *d*_*k*_ is the squared Euclidean distance between the training samples *x*_*k*_ and the input *x*.

### Evaluation Indices

To evaluate the performance and stability of the proposed model, parameters such as prediction precision, recall, and degree of integration (IoU) were defined. Evaluation of the performance of the detection and recognition model is an essential stage. The detection accuracy and complexity are the key performance indexes in the evaluation. The basic evaluation indexes of the target detection model are accuracy rate (P) and recall rate (R). The definitions of the two indicators are shown in Equations 7, 8. Accuracy rate represents the ratio that the detected target really belongs to this class, which is used to describe the credibility of the target. Recall rate represents the ratio of the detected target to the actual total amount of the target, which is used to describe the degree of the target being found completely.


(7) $$\begin{array}{l} P=\frac{T_{P}}{T_{P}+F_{P}} \end{array}$$

(8) $$\begin{array}{l} R=\frac{T_{P}}{T_{P}+F_{N}} \end{array}$$

*T*_*P*_–The number of correctly predicted targets

*F*_*P*_–The number of wrongly predicted targets

*F*_*N*_–The number of missed predicted targets

Mean average precision is used to measure the overall effect of multi-classification detection by averaging the detection precision of all categories. Its definition is shown in Formula 3.


(9) $$\begin{array}{l} MAP=\frac{\sum_{k=1}^{N}P\left(k\right)}{N} \end{array}$$

Intersection over Union (IoU) is introduced to measure the similarity between the prediction box and real box according to the characteristics of the target detection task. Its definition is shown in Formula 4. When the prediction box is exactly consistent with the real box, IoU is 1. Generally, the target is considered successfully detected when IoU is >0.5.


(10) $$\begin{array}{l} IoU=\frac{S_{A}\cap S_{B}}{S_{A}\cup S_{B}} \end{array}$$

Soybean detection and recognition is a dichotomous problem, which only involves foreground soybean and background soybean. Therefore, *F*_1_ was introduced to evaluate the model accuracy comprehensively. The value of *F*_1_ depends on the accuracy and recall. Its definition is shown in Equation 11.


(11) $$\begin{array}{l} F_{1}=\frac{2PR}{P+R} \end{array}$$

The running speed of the detection and recognition algorithm is also an important basis for model evaluation. FPS was adopted as the evaluation standard in this experiment. Its definition is shown in Equation 12. In Equation 12, N represents the total number of samples and T represents the running time.


(12) $$\begin{array}{l} FPS=\frac{N}{T} \end{array}$$

## Results and Discussion

### Soybean Leaves and Pod Types Recognition Results

The performance of popular existing algorithms such as Faster R-CNN, FPN, SSD, and YOLOv3 in soybean pod detection tasks is shown in Table 1.

**Table 1:** Soybean pod detection performance of different models.

**Model**		**Accuracy P**	**Recall R**	***F*_1_ value**	**Speed**
Existing	Faster R-CNN	86.2%	80.5%	83.3%	13FPS
algorithms	FPN	**89.8%**	82.7%	86.1%	7FPS
	SSD	80.1%	74.2%	77.0%	24FPS
	YOLOv3	87.4%	81.6%	84.4%	**39FPS**
Improved YOLOv3		**90.3%**	**87.6%**	**88.9%**	36FPS

*The bold values are the best results*.

The table indicates that FPN showed obvious advantages over the existing popular algorithms in prediction accuracy and *F*1 index up to 89.8% and 86.1% due to its relatively large structure, followed by YOLOv3, Faster R-CNN, and SSD with 87.4, 86.2, and 80.1% in accuracy and 84.4%, 83.3, and 77.0 in *F*1, respectively. YOLOv3 had the fastest speed at 39 FPS, followed by SSD, Faster R-CNN, and FPN with 24 FPS, 13 FPS, and 7 FPS, respectively. Among them, the speed of YOLOv3 was more than five times that of FPN, but the accuracy was 2.4% lower. Therefore, YOLOv3 was the best algorithm considering the prediction accuracy and speed comprehensively.

The accuracy of the improved YOLOv3 algorithm was 3.32% higher than that of YOLOv3, reaching 90.3%, and also better than that of FPN, SSD, and Faster R-CNN. The speed was second only to YOLOv3, but significantly faster than SSD, Faster R-CNN, and FPN, reaching 36 FPS, which can meet the demand of real-time recognition.

Moreover, considering that the number of seeds in different pods varied greatly, the pods were further classified into single seed pods, double seeds pods, treble seeds pods, four seeds pods, and five seeds pods according to the number of internal seeds using the above proposed YOLOv3 model.

**Figure 7:**
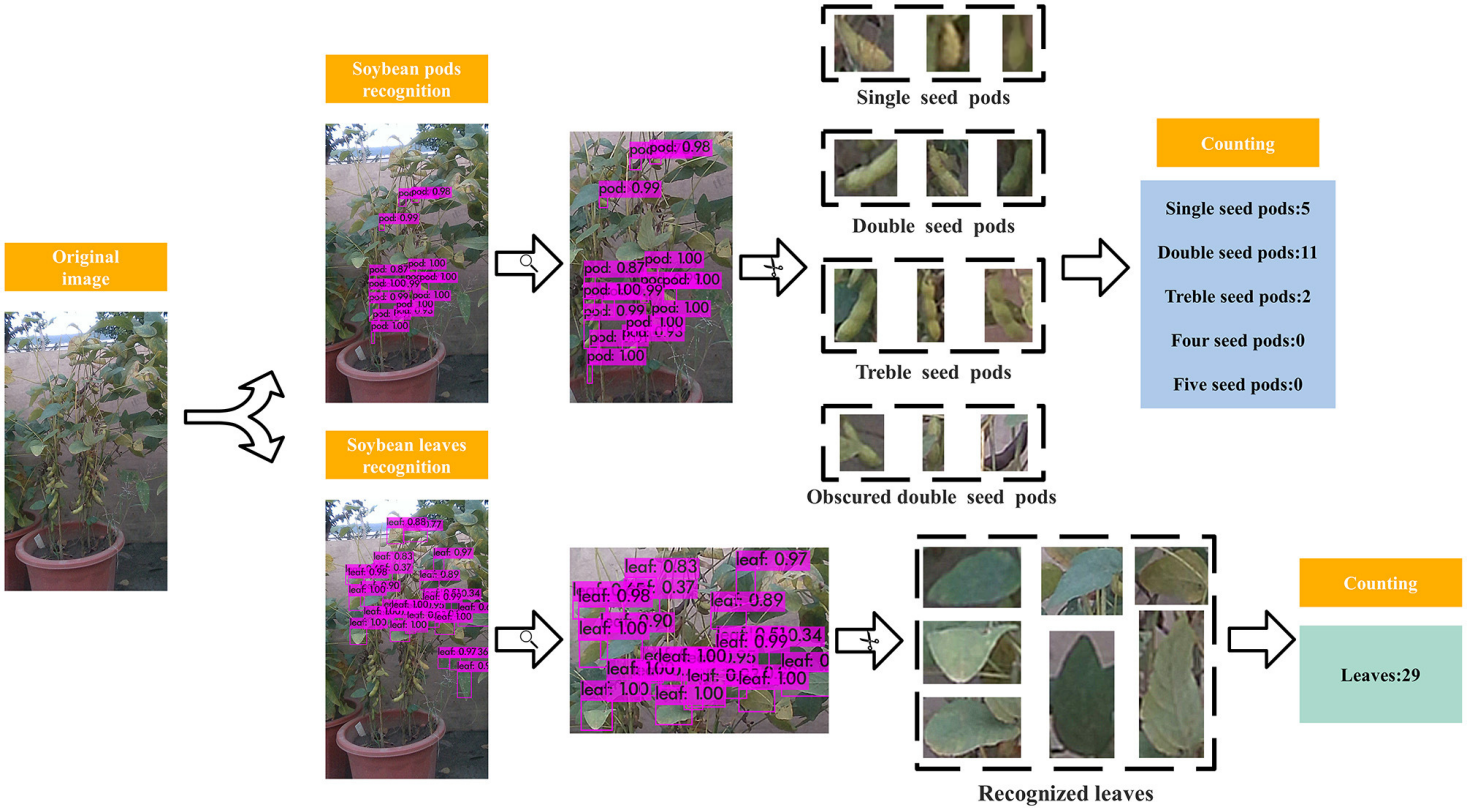
Soybean leaves and different type pods counting process.

In order to accurately identify the pod type, pod type labeling and training were carried out on the recognized pod output anchor frame, and an accurate pod type recognition model was obtained. Soybean leaves and different type pods recognition and the counting process is shown in Figure 7.

### Soybean Plant Yield Prediction Results

**Figure 8:**
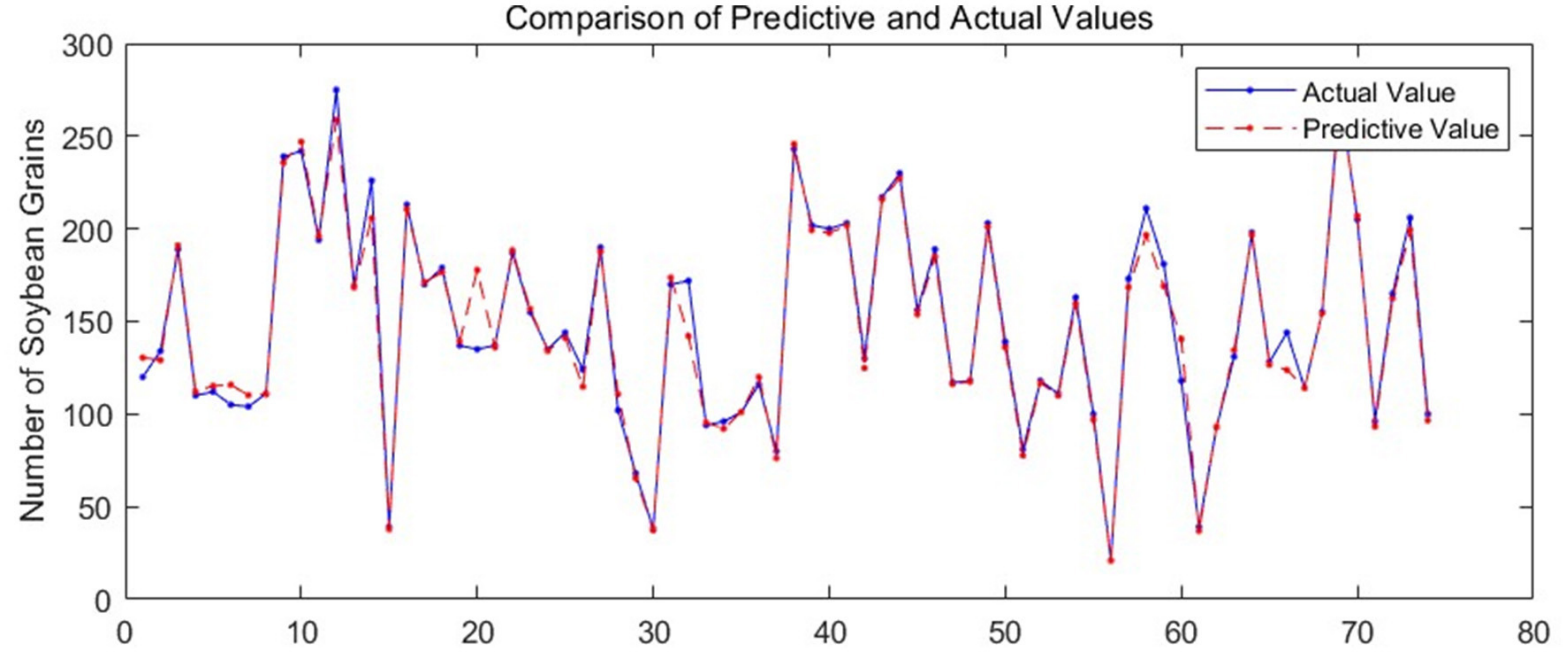
Soybean seeds number prediction results of different plants.

In the prediction of soybean yield, taking the non-uniform characteristics of leaves growth into account, four pictures were taken from four directions of soybean plants at 90 degrees apart, and the total number of leaves and the total number of different types of pods of the four images were extracted by the above mentioned improved YOLOv3 algorithm, and then the seed number prediction models of PLSR, BPNN, and GRNN were established with the input of leaf number and different type pods number, of which the results shown in Table 2 indicate that the GRNN model had the highest prediction accuracy. Figure 8 shows the comparison between the actual yield of soybean per pot and the predicted yield when the GRNN model was used for yield prediction. After the model was run three times, the average accuracy of the GRNN model was up to 97.31%.

**Table 2:** Prediction accuracy of different models.

**Model**	***ACC*1**	***ACC*2**	***ACC*3**	** $\bar{ACC}$ **
PLSR	95.57%	96.24%	95.76%	95.84%
BPNN	96.57%	96.97%	96.59%	96.71%
GRNN	97.24%	97.50%	97.20%	**97.31%**

*The bold values are the best results*.

As shown in Equation 13, *y*_*i*_ and $y_{i}^{\prime }$ represent the actual value and predicted value of soybean seeds respectively, and *n* stands for the number of pots of all soybeans. *ACC* is the accuracy calculated according to the deviation degree between the predicted value and the actual value of soybean seed number, which can reflect the performance of the prediction models.


(13) $$\begin{array}{l} ACC=\frac{\sum_{i=1}^{n}\left(y_{i}-\left|y_{i}-y_{i}^{\prime }\right|\right)/y_{i}}{n} \end{array}$$

To further measure the weight of soybean grains produced by plants, the average weight of 100 soybean seeds (*w*_*a*_ = 0.203 g) and the average weight of 100 soybean seeds for each pod type 5 to 10 days after soybean harvesting from random sampling were measured. Among them, the average weight of soybean grains in single seed pods *w*_1_, double grains pods *w*_2_, treble grains pods *w*_3_, four grains pods *w*_4_, and five grains pods *w*_5_ were 0.242 g, 0.207 g, 0.196 g, 0.189 g, and 0.186 g, respectively. Based on the numbers of different type pods predicted by the improved YOLOv3, the total weight of soybean grains produced was given, as shown in Table 3.

**Table 3:** Weight of single soybean grain of different pod types and total yield prediction results.

**Pod type**	**Single (*w*_1_)**	**Double (*w*_2_)**	**Treble (*w*_3_)**	**Four (*w*_4_)**	**Five (*w*_5_)**	**All types of pods (*w*_***a***_)**
Average weight per grain (g)	0.242	0.207	0.196	0.189	0.186	0.203
Total weight (g)			2668.670			2605.911
Accuracy			**97.43%**			95.14%

*The bold values are the best results*.

The accuracy of soybean yield predicted by GRNN was 95.14% with the average weight of 100 grains, while the accuracy of soybean yield predicted by GRNN with average weights of five different pods (*w*_1_, *w*_2_, *w*_3_, *w*_4_, and *w*_5_) increased to 97.43%. The results show that it is more accurate to predict soybean yield by identifying and classifying soybean pods combined with GRNN.

## Conclusion

Most of the existing crop yield prediction methods studied the impact of environmental changes on yield, but paid no attention to the actual yield prediction. Therefore, the most used field yield measurement method is still the traditional manual sampling process for statistical calculation, which is inefficient with low precision. Due to the high cost, time-consuming, and low accuracy of the traditional manual soybean yield measurement approach, this paper proposed a soybean yield *in situ* prediction method based on bean pods and leaves image recognition using a deep learning algorithm combined with a generalized regression neural network (GRNN). YOLOv3 is generally superior to Faster R-CNN, FPN, and SSD in terms of prediction accuracy and speed. Moreover, YOLOv3 was improved by changing the IoU loss function, using the anchor frame clustering algorithm, and utilizing the partial neural network structure in which recognition precision increased by 2.9% up to 90.3% at 36 FPS.

In this paper, we proposed to take four images of soybean plants at 90° intervals, and extract the total numbers of leaves and different type pods from the four images by improved YOLOv3. Then we established the prediction model of different type pods quantity of each plant using GRNN with inputs of the total numbers of leaves and different type pods recognized, in which the average accuracy increased to 97.31%, which was better than PLSR and BPNN. Furthermore, the soybean grain yield was calculated using the number and average weight of each type of pod. The prediction accuracy of the yield weight was up to 97.43%, which was better than the prediction accuracy based on the total number of grains and the average weight of different type pod grains.

This study shows that the improved YOLOv3 algorithm can be used to identify the number of leaves and different type pods and, moreover, can achieve accurate soybean yield *in situ* prediction 30–40 days in advance combined with the average weight of different soybean pods, which provides a new solution for accelerating soybean germplasm innovation and phenotypic detection of other crops.

## Data Availability Statement

The raw data supporting the conclusions of this article will be made available by the authors, without undue reservation.

## Author Contributions

WL proposed the conceptualization and methodology and wrote the paper. RD programmed the software. PN compared the performance of the algorithms. GX designed and carried out the experiments. HL and LS improved the methodology and conceived the experiments. All authors reviewed the manuscript.

## Funding

This research was funded by the National Natural Science Foundation of China (Nos. 32071896 and 31960487), the Agricultural Science and Technology Innovation Project of Jiangsu Province [No. CX(20)3068], the Modern Agricultural Machinery Equipment and Technology Demonstration and Promotion Project of Jiangsu Province (NJ2021-37), the Agricultural Science and Technology Innovation Project of Suzhou City (No. SNG2020039), and National Foreign Experts Program of China (G2021145010L).

## Conflict of Interest

The authors declare that the research was conducted in the absence of any commercial or financial relationships that could be construed as a potential conflict of interest.

## Publisher's Note

All claims expressed in this article are solely those of the authors and do not necessarily represent those of their affiliated organizations, or those of the publisher, the editors and the reviewers. Any product that may be evaluated in this article, or claim that may be made by its manufacturer, is not guaranteed or endorsed by the publisher.
